# Evaluate the Effectiveness of Enhanced Recovery After Surgery Versus Conventional Approach in Benign Gynecological Surgeries: A Randomized Controlled Trial

**DOI:** 10.7759/cureus.16527

**Published:** 2021-07-21

**Authors:** Anupama Bahadur, Payal Kumari, Rajlaxmi Mundhra, Anoosha K Ravi, Latika Chawla, Mahima Mahamood M, Purvashi Kumari, Jaya Chaturvedi

**Affiliations:** 1 Obstetrics and Gynecology, All India Institute of Medical Sciences, Rishikesh, IND

**Keywords:** quality of life, length of hospital stay, enhanced recovery after surgery, benign gynecological surgery, postoperative management

## Abstract

Objective

This study aimed to evaluate the effectiveness of enhanced recovery after surgery (ERAS) model versus conventional approach in benign gynecological surgeries (incorporating various routes of surgery).

Methods

This was a randomized controlled trial wherein patients undergoing gynecological surgery for benign indications from January 2019 to July 2020 were recruited and randomized into ERAS and conventional protocol groups using block randomization. The intended primary outcome was to compare the median length of hospital stay in both groups. “Fit for discharge” criteria were used to assess the length of stay as patients who belonged to hilly terrain with limited transportation facilities stayed for a longer duration.

Results

A total of 180 patients were recruited and 90 each was randomized into ERAS and conventional protocol groups. The difference in length of hospital stay between ERAS (36 hours, range 24-96 hours) and conventional group (72 hours, range: 24-144 hours) was significant (p<0.01). A statistically significant difference was noted in the time for recovery of bowel function and tolerance for diet in the ERAS group. No significant difference in complications and readmission (within 30 days) rate was seen between the two groups. Quality of life as assessed by the World Health Organization Quality of Life Brief Version (WHO-QOL BREF) on the day of discharge and day 30 was higher in the ERAS group in physical and psychological domains, while no difference was seen in environmental and social domains.

Conclusion

This study as an institutional experience strengthens the existing evidence regarding the efficacy of ERAS in reducing hospital stay and improving quality of life compared to the conventional perioperative management protocol.

## Introduction

Enhanced recovery after surgery (ERAS) is a multidisciplinary approach, comprehensively designed to improve postoperative outcome. In 1997, a Dutch professor Henrik Kehlet gave the concept of “multimodal approach to control postoperative pathophysiology and rehabilitation” [1]. Colorectal surgery was the first surgical subspecialty where the ERAS pathway was implemented in the year 1999 [2]. The term ERAS was given in 2001 by a team of surgeons who met in London to develop guidelines for perioperative care, grounded on evidence [3]. ERAS program is also referred to as “rapid recovery program,” “multimodal perioperative management,” or “fast-track program.” It involves the cooperation of surgeons, anesthetists, and staff caring for patients. Stress is the key pathogenic factor resulting in postoperative morbidity and organ dysfunction [4]. This knowledge has encouraged the development of techniques to ease undesirable responses. Patient counseling about surgery and postoperative recovery period, reducing the duration of preoperative fasting, technique for pain alleviation for early ambulation, control of nausea, vomiting and ileus, realizing the benefits of early enteral nutrition, and antithrombotic and antimicrobial prophylaxis are the techniques directed to early recovery [5]. On the other hand, traditional practices encourage the use of drains, nasogastric tubes, catheters, restriction of oral intake, and ambulation. Gradually, these are losing popularity as there is no scientific proof to support such a practice.

The goal of ERAS protocol is to mitigate surgery-related morbidities, reduce postoperative pain and analgesic use, complications, and readmissions rate, improve patient satisfaction, and reduce hospital stay. On account of its successful implementation in colorectal surgery and other specialties, there has been a demand for investigating ERAS in gynecological surgeries. There is limited data on the effectiveness of ERAS program in gynecological procedures, especially benign surgeries. With this aim, we conducted this study as an institutional experience to compare the postoperative outcome in ERAS versus conventional protocol in benign gynecologic surgeries through robot-assisted, laparoscopic, abdominal, and vaginal routes. This study is unique in two aspects. Firstly, it assesses the outcome of various types of surgeries including robotic surgeries. Secondly, this study is the first of its kind from Himalayan terrain with poor transportation facilities.

## Materials and methods

This was a single-center randomized controlled trial conducted in the Department of Obstetrics and Gynecology from January 2019 to July 2020 after approval from the Institutional Ethics Committee (AIIMS/IEC/19/829). The study was registered with the Clinical Trial Registry of India: CTRI/2019/11/021959.

**Figure 1:**
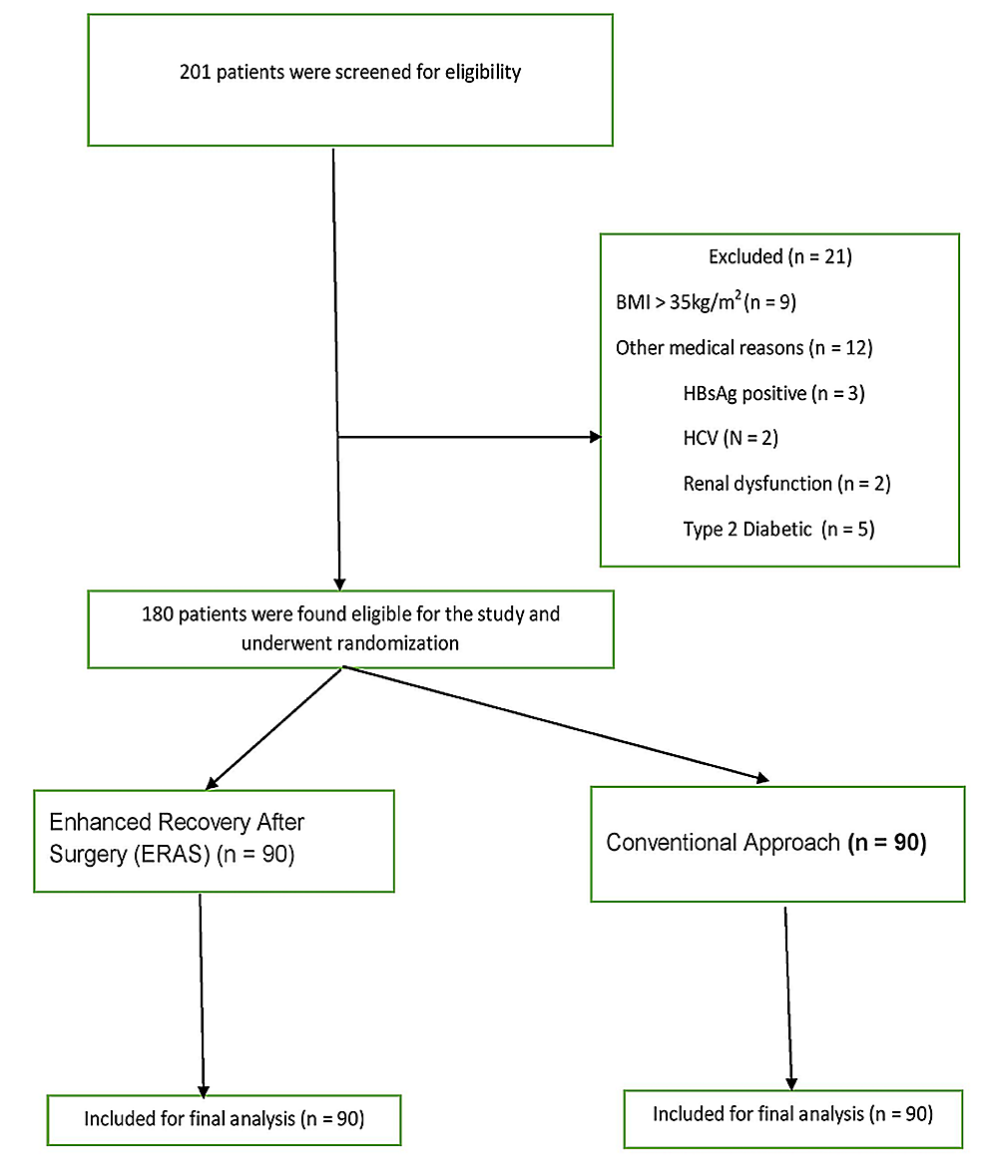
CONSORT flow diagram CONSORT: Consolidated Standards of Reporting Trials

The sample size required in each arm of the study was calculated according to the formula 2(zα + z1-β ) 2 σ 2 /δ 2. The sample size for the study was based on a study by Kalogera et al. [6]. The mean duration of hospital stay in the ERAS arm in this study was 2.7 days (SD 0.8) while that in the convectional arm was 3.2 days (SD 0.9). Thus, considering the difference of means (δ ) of 0.5 and pooled SD (σ) of 0.85 and assuming 95% power and 95% confidence interval, 80 patients in each arm would be needed for the study. To allow for 10% attrition, 90 patients were taken in each arm (Figure 1).

Patients undergoing gynecological surgeries for benign indications (cases with fibroid uterus, adenomyosis, prolapse uterus, fibroid polyp, etc.) through robot-assisted laparoscopy, open or vaginal routes were assessed for eligibility. The route of surgery was decided as per the indication of surgery. Detailed history followed by physical examination including general and systemic examination was done for all patients. Patients with BMI>35 kg/m^2^ were not included in the study. Obese patients were excluded because generally surgeries are prolonged with a higher risk of postoperative complications in such patients. The primary outcome of the study was to compare the postoperative median length of hospital stay between the two groups. “Fit for discharge” criteria were used to assess the length of stay, as patients who belonged to hilly terrain with limited transportation facilities stayed for a longer duration. Discharge was at the surgeon’s discretion. Secondary outcomes were to note time to tolerance of diet (in days), time to pass flatus/defecation (in days), postoperative complications, readmission rate within 30 days of discharge, and quality of life by World Health Organization Quality of Life Brief Version (WHO-QOL BREF) [7].

After taking written informed consent from the participants, they were randomized into two groups by block randomization method irrespective of the route of the surgery using SPSS software IBM version 16 (Armonk, NY: IBM Corp.). We used a block of four in this study. One group was managed using the ERAS protocol and the other was managed using conventional perioperative management protocol that was routinely followed at the study institution (Table 1).

**Table 1:** Perioperative management protocol offered to ERAS vs. conventional group ERAS: enhanced recovery after surgery

	ERAS Protocol	Conventional Protocol
Patient education	Patient counseled regarding surgery, postoperative pain, and other morbidities if associated, which helped in alleviating anxiety related to surgery	Patient counseled regarding the surgical procedure and its immediate and late complications
Bowel preparation	No mechanical bowel preparation. Oral bowel preparation was done with Neomycin 1 gram orally thrice daily and metronidazole 500 mg orally thrice daily, a day prior to surgery	Liquid diet a day prior to surgery. Mechanical bowel preparation with peglec sachet in 2 liters of water from 12 noon. Oral preparation by ampicillin 500 mg orally 4 times a day and rectal enema at bedtime and on the morning of surgery
Preoperative diet	Solid food allowed up to 6 to 8 hours before the procedure. Carbohydrate-rich fluid (50 grams glucose in 200 ml water) - 4 hours before surgery. Clear fluids (water, apple juice) up to 2 hours before surgery	Evening before surgery - solids until 10 pm. Nil per oral after 10 pm
Preoperative medications	Preemptive analgesia with acetaminophen 1000 mg oral/Intravenous (IV) once on morning of surgery. Bath or shower with betadine scrub/soap or antiseptic agent the night before surgery	Injection pantoprazole 40 mg IV. Bath or shower with soap or antiseptic agent the night before surgery
Antimicrobial	Chlorhexidine–alcohol for skin cleansing. Injection ceftriaxone 1 gram IV 30 minutes before incision	Chlorhexidine–alcohol for skin cleansing. Injection ceftriaxone 1 gram IV 30 minutes before skin incision. Injection metronidazole 500 mg IV 60 minutes before skin incision
Antiemetic	Postoperative nausea and vomiting prophylaxis using ≥2 antiemetics. Injection dexamethasone 4mg IV at induction. Injection ondansetron 4 mg IV at the end of surgery and 8 hourly	Injection dexamethasone 4mg IV at induction. Injection ondansetron 4mg IV before anesthesia. Injection metoclopramide 10 mg IV 8 hourly after surgery
Anesthesia	Long-acting opioid and sedating agent was avoided. Spinal and epidural anesthesia as indicated. Maintenance of normothermia	General anesthesia, spinal anesthesia, combined epidural and spinal anesthesia as indicated
Drains and catheters	Avoidance of drains and nasogastric tubes. Foley’s catheter removal on postoperative day (POD) 1 except in cases of bladder injury	Surgical drain if indicated. Removal of Foley’s catheter-based on intraoperative findings
Fluid therapy	Goal-directed. Very restrictive or liberal fluid regimes were avoided	Fluids at 100 mL/hr for 12–24 hours or till oral liquid was tolerated. Fluid bolus of 250–500 mL for urine output less than 30 mL/hr was given
Postoperative optimization	Semisolid diet started POD 0 followed by solid the next day. Chewing gum orally for 30 minutes after meals 3 times per day as tolerated starting on POD 0. Maintenance of normothermia	Nil per oral status at surgeon’s discretion followed by oral sips of clear liquid and semisolids
Analgesia (multimodal)	Transversus abdominis plane block wherever indicated. Injection paracetamol 1000 mg IV 6 hourly. Injection diclofenac 75 mg IV 8 hourly (reserve analgesia if visual analog scale >4). Acetaminophen 1 gram per oral 6 hourly (should not exceed 4000 mg/24 h from all sources) (started on POD 1). Diclofenac 50 mg per oral 8 hourly started on POD 1. Tramadol 100 mg per oral 4-6 hourly started on POD 1	Injection paracetamol 1 gram IV 6 hourly. Injection diclofenac 75 mg IV 8 hourly. Injection tramadol 50-100 mg 6 hourly. Injection morphine 2 mg IV (if not controlled by above 3). Oral analgesics started when patient tolerated diet
Activity	Evening of surgery: out of bed greater than 2 hours including one or more walks and sitting in chair. Day after surgery and until discharge: out of bed greater than 8 hours including four or more walks and sitting in chair. Patient up in chair for all meals	Ambulation as soon as possible when patient was comfortable
Venous thromboembolism (VTE) prophylaxis	Low molecular weight heparin started POD 1 as and when indicated. Sequential compression devices while in bed in hospital as and when indicated	Low molecular weight heparin started POD 1 when indicated (prolonged surgery)

Quality of life assessment was done on the day of discharge and day 30. The questionnaire assessed four domains (physical, psychological, social, and environmental) with a total of 26 questions. The four domain score denoted an individual’s perception of quality of life in that particular domain. The physical domain assessed energy, fatigue, sleep, daily activities, pain, and work capacity. The psychological domain assessed feelings, self-esteem, memory, concentration, etc. The social domain was for social support from family and friends, and the environmental domain was for financial security, health care accessibility, and other surrounding factors. Domain scores were scaled in a positive direction (i.e., higher scores denoted higher quality of life). The mean score of items within each domain was used to calculate the domain score. Raw scores were calculated and transformed to “transformed score 4-20” using SPSS syntax software (Armonk NY: IBM Corp.), which was comparable to WHO-QOL 100. The mean score in each domain was calculated for ERAS group and compared with the conventional group.

Statistical analysis

Data analysis was done using SPSS package IBM version 21 (Armonk NY: IBM Corp.). Continuous variables were tested for normality assumption using an appropriate statistical test. Descriptive statistics such as mean, standard deviation, and deranged values were calculated for normally distributed data. Comparison of mean values between the two groups was done using Student’s t-test. For skewed data, median and interquartile range were calculated and comparison of median values was done using non-parametric Mann-Whitney U test. Categorical variables were presented as frequency and percentage values. Comparison of frequency across categories was done using chi-square test/Fischer’s exact test as appropriate. For all the statistical tests, a two-sided probability of p<0.05 was considered for statistical significance. All p-values were derived with a 95% confidence interval.

## Results

The baseline characteristics were comparable between the two groups (Table 2). Table 3 shows the distribution of route of surgery among the two groups.

**Table 2:** Baseline characteristics ERAS: enhanced recovery after surgery

S. No.	Parameter		ERAS (n=90)	Conventional (n=90)	p-Value
1	Age (years)	Mean±SD	43.5±9.1	43.3±7.8	0.41
		Range	20-60	23-68	
2	Weight (kg)	Mean±SD	62±7.7	61.4±8.8	0.27
		Range	54-88	45-86	
3	Height (cm)	Mean±SD	154.7±6.7	155.6±6.7	0.25
		Range	140-169	140-166	
4	BMI (kg/m^2^)	Mean±SD	25.6±3.4	25.2±3.4	0.33
		Range	20-34	20.4-33.5	
5	Parity	Median	3	3	0.08
		Range	0-7	0-8	

**Table 3:** Route of surgery Data expressed as n% ERAS: enhanced recovery after surgery

Procedure	ERAS (n=90)	Conventional (n=90)
Robot-assisted	44.44% (n=40)	44.44% (n=40)
Laparoscopic	11.11% (n=10)	11.11% (n=10)
Abdominal	22.22% (n=20)	22.22% (n=20)
Vaginal	22.22% (n=20)	22.22% (n=20)

Table 4 shows the comparison of the postoperative outcomes between the two groups. A significant difference (p<0.01) was seen in the length of hospital stay in the ERAS group compared to the conventional group. In the ERAS group, the length of hospital stay was 24 (24-36), 36 (24-48), 56 (48-72), and 48 (24-96) hours, and in the conventional group, it was 48 (36-72), 48 (24-72), 72 (48-144), and 72 (48-120) hours for robotic, laparoscopic, abdominal, and vaginal routes, respectively. ERAS group was noted to have earlier return of bowel function as evident by earlier passage of flatus and feces. Time to tolerance for diet was also sooner in the ERAS group with almost 92% of patients starting on oral feeds from the postoperative day zero (POD 0). In the conventional group, oral feeds were started as per the surgeon’s discretion and it was mostly on POD 1. No significant difference was seen in postoperative complications and readmission rates between the two groups. One patient who had urinary retention in the ERAS group was managed by catheterization of the bladder for few hours. None of the patients in either group had venous thromboembolism. One patient in the ERAS group, who underwent laparoscopic surgery got readmitted. Whereas six patients in the conventional group were readmitted within 30 days of discharge of which three had undergone robotic surgery, one had abdominal surgery, one had vaginal surgery, and one had laparoscopic surgery.

**Table 4:** Comparison of postoperative outcome between the two groups *Statistically significant at p<0.05. Data expressed as mean±SD (range) or n (%) POD: postoperative day; NA: not applicable; ERAS: enhanced recovery after surgery

Outcome	ERAS (n=90)	Conventional (n=90)	p-Value
Length of hospital stay (Fit for discharge criteria), hours	39.2±17.72 (24-96)	62.95±19.5 (24-144)	<0.01*
Length of hospital stay, hours	51.33±25.7 (24-120)	73.97±24.04 (24-144)	<0.01*
Time to tolerance for diet			<0.01*
POD 0	83 (92.2%)	2 (2.2%)	
POD 1	6 (6.6%)	82 (92.1%)	
POD 2	1 (1.1%)	6 (6.6%)	
Time to passage of flatus			<0.01*
POD 0	56 (62.2%)	14 (15.56%)	
POD 1	33 (36.67%)	72 (80.00%)	
POD 2	1 (1.1%)	4 (4.4%)	
Time to defecation			<0.01*
POD 0	81 (90%)	6 (6.67%)	
POD 1	8 (8.89%)	74 (82.2%)	
POD 2	1 (1.1%)	10 (11.1%)	
Complications			
1. Fever	1 (1.11%)	6 (6.67%)	0.053
2. Gastrointestinal			
Nausea/vomiting	6 (6.6%)	4 (4.4%)	0.51
Paralytic ileus	1 (1.1%)	1 (1.1%)	1
3. Urinary tract			
Infection	1 (1.1%)	1 (1.1%)	1
Retention	1 (1.1%)	0	NA
4. Wound			
Surgical site infection	0	2 (2.2%)	NA
Dehiscence	0	1 (1.1%)	NA
5. Vault			
Discharge per vagina	1 (1.1%)	3 (3.33%)	0.31
Readmission	1 (1.11%)	6 (6.66%)	0.053

When the quality of life was assessed by using the WHO-quality of life BREF questionnaire, a significant difference in physical (p<0.01) and psychological domains (p<0.01) was seen both on the day of discharge and on day 30 in the ERAS group (Table 5). No difference was seen between the two groups in social or environmental domains.

**Table 5:** Quality of life assessment in both groups *Statistically significant at p<0.05. Data expressed as mean±SD ERAS: enhanced recovery after surgery

	ERAS (n=90)	Conventional (n=90)	p-Value
On day of discharge			
Physical domain	14.2±0.56	13.5±0.56	<0.01*
Psychological domain	15±0.37	13.9±0.80	<0.01*
Social domain	10±1.05	10.3±1.01	0.07
Environmental domain	16.8±0.69	16.8±0.72	0.5
On day 30			
Physical domain	16.35±0.84	13.5±0.93	<0.01*
Psychological domain	16.55±0.90	14.8±1.25	<0.01*
Social domain	16.8±0.93	16.8±0.89	0.43
Environmental domain	16.9±0.73	16.9±0.51	0.4

## Discussion

The conventional approach to postoperative management has been in use for many years may be just as a practice of habit without any scientific basis. ERAS protocol claimed to be better in comparison to the conventional approach as reported from other specialty surgeries. The authors intended to find the basis of this result with respect to benign gynecological surgeries. A benefit in terms of most of the intended outcomes was found with the ERAS protocol in the present study. The discussion is based on the similarity of such a finding in studies done with different varieties of samples. Thus, it was concluded that ERAS which has found a better outcome than the irrational conventional approach is recommended. This was also an opportunity to introduce this advancement into the routine protocol of perioperative management and educate the personnel involved in perioperative care. The benefit of lesser hospital stay found in the present study was consistent with studies conducted by Wijk et al., Mukhopadhyay and Khalil, and Modesitt et al., where patients receiving ERAS protocol were discharged earlier [8-10].

Wijk et al. observed in their study that patient passed flatus on day one (0-10) in preERAS (n=120) and day one (0-3) in ERAS (n=85) [8]. A similar result was noted in the present study wherein the bowel function recovered sooner in the ERAS group.

No significant difference was found in complications or readmission rate between the two groups, and the patients who got readmitted did not favor one route of a surgery over the other in the present study. A similar observation was found in the literature [11,12]. Yoong et al. in their study on rapid recovery program in vaginal hysterectomy patients observed that 4% of patients were readmitted before and 0% after execution of an ERAS program, but the difference was not significant (p>0.05%) [11]. Relph et al. also reported no difference in readmission rate in their case-control study of vaginal hysterectomy patients [12].

A better quality of life in physical and psychological domains was noted in the ERAS group with no difference in social and environmental domains. Similarly, Yoong et al. assessed patient satisfaction level with the ERAS program on the scale of 1 to 10, in patients of vaginal hysterectomy before (n = 50) or after (n = 50) ERAS implementation (after four weeks of surgery) [11]. The authors observed that the median satisfaction score was 8/10 in both groups and 65% of patients in the ERAS group gave scores of greater than 9/10. Philp et al., in 2014, assessed patient satisfaction in a fast-track setting using in-patient satisfaction with care measure (INPATSAT-32) questionnaire that was mailed out one month after surgery [13]. A total of 96% of patients indicated “good to excellent” for coordination of care from diagnosis to discharge and 92% “good to excellent” for efficiency of nursing care.

Thus, ERAS protocol is a beneficial approach to perioperative care in patients undergoing gynecological surgery for benign indications irrespective of the route of surgery. Though the protocol entailed some drastic changes over the conventional approach, the implementation into the routine functioning in the present scenario was not challenging and the benefits observed definitely made the continuation of usage a reality.

The findings of the study are subject to these limitations. First, though the groups were comparable in the choice of route of surgery, a majority of patients underwent robot-assisted surgery (80/180). As robot-assisted surgeries generally have the benefit of faster recovery and earlier discharge, the final result could have been influenced by this. Second, the indications for the surgery were not a part of the selection process which also could have been the source of bias for the observations in postoperative outcome. Third, a smaller sample size. Thus, further studies evaluating individual route of surgery and/or indication with a larger sample size are recommended before generalizing the findings of this study.

## Conclusions

The effectiveness of ERAS is based on its ability to break the stress cascade while maintaining the normal physiology as far as possible before and after surgery. Preoperative patient education prepares the patient for early discharge. Ambulating the patient early, starting feeds early, removing Foley catheter early, anti-emetics and multi-modal analgesia at all stages of care contribute to the patient being discharged from the hospital earlier than what is followed conventionally.

The present study strengthens the existing evidence that when successfully implemented, ERAS program leads to a faster recovery and earlier discharge and ultimately improved quality of life and patient satisfaction. Patients in ERAS group tolerated diet well on the day of surgery and had earlier return of bowel function. The readmission rate was also observed to be less in ERAS group. Its effectiveness is not limited by the route of surgery as length of stay decreased in minimally invasive, abdominal as well as vaginal surgeries when assessed individually in our study. Its effect on quality of life is independent of the route of surgery and is found to be better in ERAS group.

Though there are many studies assessing its impact in gynecologic oncology surgeries, further studies are required in the field of gynecological surgeries for benign indications especially minimally invasive.
